# St. Bartholomew's Hospital

**Published:** 1904-07-09

**Authors:** 


### ST. BARTHOLOMEW'S HOSPITAL.

### FOUNDATION-STONE OF THE NEW BUILDINGS LAID BY THE KING.

Magnificent weather, brilliant sunshine tempered by pleasant breezes, favoured the visit of the King and Queen on Wednesday (6th instant) to St. Bartholomew's Hospital to lay the foundation-stone of the new wing. Their Majesties were greeted by cheering crowds, as, accompanied by a glittering escort of Household Cavalry, they drove through the City, a momentary pause being made at the boundary in Holborn, when the Lord Mayor, surrounded by a scarlet-clad group of civic officials, surrendered the historic pearl sword to his Majesty. On the new ground a great marquee, with tier above tier of seats in the form of an amphitheatre, to hold 3,000 guests, had been erected, and this was filled to its utmost capacity. Among the guests who had accepted invitations to be present were the Presidents of the Royal Colleges of Physicians and of Surgeons, the staff of the hospital, and many other representatives of medical science; the Bishop of London, the Archdeacon of London, and the Chief Rabbi; the Lord Mayor and Corporation of the City; the London members of Parliament and the mayors of the metropolitan boroughs; the Masters of the great Livery Companies; the Governor of the Bank of England; and Sir Charles Wyndham, Mr. Beerbohm Tree, Mr. George Alexander, Mr. Edward Terry, and other leading lights of the dramatic world. The band of the Coldstream Guards discoursed sweet music, while the bright dresses of the ladies, the many aldermanic and academic robes, with the more sober uniforms of the nurses, made the scene a gay and notable one.

The Prince of Wales, the president of the Hospital, with the Princess and suite, arrived early in company with Sir Trevor Lawrence, the treasurer; Sir Ernest Flower, the secretary; and Sir William Treloar, the chairman, of the Special Appeal Committee. Soon afterwards a burst of cheering from the crowd outside announced the arrival of their Majesties. The King wore the uniform of a Field Marshal, while the Queen was radiant in heliotrope and pearls. As they entered, the great audience rose to the sound of the National Anthem, and after the presentation of lovely bouquets to the Royal ladies by the matron (Miss Isla Stewart) and the treasurer, the Bishop of London opened the proceedings with a prayer.

The Prince of Wales, as President, then read the following address:—

To the King's and Queen's Most Excellent Majesties.

May it please your Majesties,

As President of the Royal Hospital of St. Bartholomew, and on behalf of the Governors of that ancient foundation, I desire to convey to your Majesties, with our loyal duty, our attachment and devotion to your persons and to the Throne. I have to express our very grateful appreciation of the warm and unfailing interest your Majesties have ever shown in the welfare and progress of the institution, as well as our deep sense of the honour your Majesties have done the Hospital in consenting to lay the foundation-stone of its new buildings, especially as her Majesty the Queen is our first Lady Governor and the first donor to our Appeal Fund.

Founded in the year 1123, St. Bartholomew's has carried on a great work of mercy and charity on the same spot for close upon 800 years. It has had its vicissitudes; but, thanks in great measure to the favour of 11 of his Majesty's predecessors on the Throne, it has maintained its position, and overcome all difficulties. Since the Charter granted by King Henry VIII. in the year 1547 the record of the Hospital has been one of tranquillity and progress, rendered remarkable, however, by the connection with it of many names illustrious in the history and advance of medical science.

At the present day St. Bartholomew's contains 740 beds, inclusive of 70 in its country branch at Swanley. During the last 50 years the hospital has given gratuitous relief to more than 7,000,000 of the sick poor. The students working daily within its walls average about 500, and in the great school attached to it more than 6,000 students have received medical education during the last half-century, and have carried the fame of their "alma mater" all over the world.

The rapid advances of medical science during recent years have compelled us, acting on the advice of our eminent Medical Staff, to contemplate the entire rebuilding of the hospital as funds may become available. With the sanction of your Majesty, our President for 34 years, we have issued an appeal to the public to enable us to carry out the rebuildings referred to. To the City of London and its wealthy citizens this appeal should come home with peculiar force, for the hospital in its present development is in great measure the creation of the inhabitants of what has been termed the "wealthiest square mile in the world."

We are most anxious to avoid the necessity of adding one more to the innumerable annual appeals which beset the charitable. This we hope to be able to do if the public will help us to carry out the necessary rebuildings and additions. St. Bartholomew's has not asked for help for more than 150 years, and has now provided a large sum out of its own resources for the inevitable extension of the Hospital.

We, the Governors, in again most earnestly thanking your Majesties for your presence here to-day, trust that it may be regarded by the public as showing your Majesties' desire that the rebuilding of this great and ancient institution may not be impeded and crippled for lack of funds.

To this his Majesty replied: The Queen and I have great pleasure in being present to-day to lay the foundation-stone of the new building of this famous Hospital, and we thank you heartily for the loyal address which our dear son, as President, has presented on behalf of the governing body.

You have reminded us of the antiquity of this foundation, and of the favour shown to it by so many of our royal predecessors.

I recollect the lively satisfaction which I felt in holding the Presidency of this Hospital for many years. I shall continue to take the deepest personal interest in its work and fortunes.

We are happy to see that those who are responsible for carrying on the excellent work have shown by their scheme for rebuilding the Hospital their intention that the future of St. Bartholomew's shall not be unworthy of its fine traditions in the past.

The names of the famous men educated here prove that an institution of this kind is not merely a refuge for suffering but a school for the advancement of the science and practice of medicine, and in this way the influence of this and the other great hospitals of this country extends beyond merely local limits to foreign countries and to the colonies and dependencies of our Empire.

We confidently believe that our subjects, and especially the citizens of London, will not fail to show interest in the progress of the scheme for rebuilding the Hospital nor allow that beneficent undertaking to be hindered by want of the necessary funds.

This speech, as well as the Prince's address, was received with cheers.

Sir Trevor Lawrence then formally asked his Majesty to be pleased to lay the foundation-stone; and after the architect, Mr. E. B. I'Anson, had submitted the plans of the proposed buildings, and handed to his Majesty the specially designed trowel and mallet presented by the Bromsgrove Guild, the great block of polished granite was lowered into position: and the King, having bestowed the customary three Masonic taps upon it, declared it, in the name of the Trinity, to be "well and truly laid."

The inscription on the stone is as follows:—

This Foundation Stone
of the
New Buildings of St. Bartholomew's Hospital
was laid on
6th July, 1904,
by
EDWARD VII.
King of Great Britain and Ireland
and of
The British Dominions beyond the Seas,
Defender of the Faith,
Emperor of India,
near the Site given in
1123
by
HENRY I.,
King of the English,
and ever since
devoted to the Relief of Pain and the Cure of
Disease
Among the Poor of London;
and, through the Increase of Knowledge
in the Medical Art
here attained,
to the Alleviation of Human Suffering
throughout the World.

Fear God. Honour the King.

After this ceremony the King announced his intention of giving £1,000 to the building fund; and then the Queen, the first lady elected a Governor of the Hospital, as well as the first donor to the building fund, was charged by the clerk of the Hospital, in accordance with ancient custom, in the following terms:—

"Your Majesty having been elected and chosen a Governor of St. Bartholomew's Hospital, it is your duty and charge to acquit yourself in that office with all faithfulness and sincerity; endeavouring that the affairs and business of the said Hospital may be well ordered and managed; and promoting the weal and advantage of the poor wounded, sick, maimed, diseased persons harboured in the said Hospital.

"To this end your Majesty is now admitted a Governor of St. Bartholomew's Hospital."

A copy of this charge and a Governor's staff were then handed to her Majesty, after which Madame Albani, assisted by Miss Katharine Jones, Mr. Archdeacon, Mr. Binyon, and (at the piano) Mr. Theodore Flint, sang Sir Edward Elgar's arrangement of "God Save the King," Madame Albani being presented with a bouquet on behalf of the students.

This concluded the official proceedings, but while the Royal party were being driven away to the sound of further cheering, Madame Albani delighted the audience by singing "Home, Sweet Home," not even the canvas roof of the great marquee serving to impair the tones of her wonderful voice.

